# Vascular Dysfunction in Preeclampsia

**DOI:** 10.3390/cells10113055

**Published:** 2021-11-06

**Authors:** Megan A. Opichka, Matthew W. Rappelt, David D. Gutterman, Justin L. Grobe, Jennifer J. McIntosh

**Affiliations:** 1 Department of Physiology, Medical College of Wisconsin, Milwaukee, WI 53226, USA; mopichka@mcw.edu (M.A.O.); dgutt@mcw.edu (D.D.G.); jgrobe@mcw.edu (J.L.G.); 2 Cardiovascular Center, Medical College of Wisconsin, Milwaukee, WI 53226, USA; matthewrappelt@gmail.com; 3 Department of Medicine, Medical College of Wisconsin, Milwaukee, WI 53226, USA; 4 Neuroscience Research Center, Medical College of Wisconsin, Milwaukee, WI 53226, USA; 5 Comprehensive Rodent Metabolic Phenotyping Core, Medical College of Wisconsin, Milwaukee, WI 53226, USA; 6 Department of Biomedical Engineering, Medical College of Wisconsin, Milwaukee, WI 53226, USA; 7 Department of Obstetrics and Gynecology, Medical College of Wisconsin, Milwaukee, WI 53226, USA

**Keywords:** preeclampsia, pregnancy, gestation, hypertension, vessel, blood pressure, placenta, trophoblast

## Abstract

Preeclampsia is a life-threatening pregnancy-associated cardiovascular disorder characterized by hypertension and proteinuria at 20 weeks of gestation. Though its exact underlying cause is not precisely defined and likely heterogenous, a plethora of research indicates that in some women with preeclampsia, both maternal and placental vascular dysfunction plays a role in the pathogenesis and can persist into the postpartum period. Potential abnormalities include impaired placentation, incomplete spiral artery remodeling, and endothelial damage, which are further propagated by immune factors, mitochondrial stress, and an imbalance of pro- and antiangiogenic substances. While the field has progressed, current gaps in knowledge include detailed initial molecular mechanisms and effective treatment options. Newfound evidence indicates that vasopressin is an early mediator and biomarker of the disorder, and promising future therapeutic avenues include mitigating mitochondrial dysfunction, excess oxidative stress, and the resulting inflammatory state. In this review, we provide a detailed overview of vascular defects present during preeclampsia and connect well-established notions to newer discoveries at the molecular, cellular, and whole-organism levels.

## 1. Introduction

Preeclampsia is a pregnancy-related hypertensive disorder and a major cause of maternal and perinatal morbidity and mortality [1,2]. Despite its prevalence, well-cataloged risk factors, and clinical characteristics, the exact pathophysiology of this disorder remains unknown [2]. This deficit in knowledge has hampered the development of targeted therapies and limited treatment options for healthcare providers.

**Figure 1:**
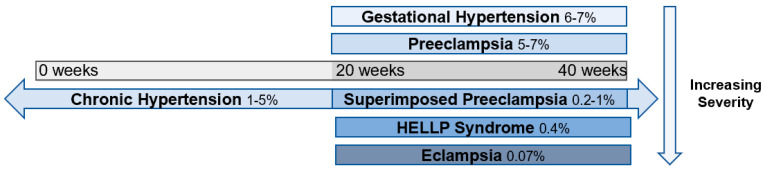
Spectrum of hypertensive disorders during pregnancy and their prevalence [9,10,11]. Gestational hypertension is defined by new-onset elevations in blood pressure (<140/90 mmHg) after 20 weeks of gestation, whereas preeclampsia is also accompanied by proteinuria and end-organ dysfunction. Chronic hypertension is present prior to 20 weeks of gestation or continues >12 weeks into the postnatal period and can occur in concert with preeclampsia. Hemolysis, elevated liver enzymes, and low platelets (HELLP) syndrome is classified as a subset of preeclampsia, and eclampsia is a complication of preeclampsia characterized by the addition of seizures.

Clinically, preeclampsia is associated with a number of complications for both mother and fetus [3]. It is thought to lie on a spectrum of hypertensive diseases in pregnancy, with gestational hypertension at the mildest end of the spectrum, followed by preeclampsia, chronic hypertension with superimposed preeclampsia, hemolysis, elevated liver enzymes, low platelet count (HELLP) syndrome, and eclampsia at the most extreme end [1] (Figure 1). As preeclampsia is a true systemic disease, it may manifest in a number of different ways. Classically, it has been defined as maternal hypertension and renal dysfunction, specifically characterized by proteinuria [1]. However, more recent guidelines have noted thrombocytopenia, impaired liver function, pulmonary edema, and cerebral/visual symptoms as diagnostic features [1,2,4]. Additional maternal complications include seizures (eclampsia), cerebral hemorrhage, disseminated intravascular coagulation, and hepatic rupture [4]. Obstetric complications associated with preeclampsia consist of uteroplacental insufficiency, placental abruption, prematurity, and increased risk of cesarean delivery [2]. Additional fetal complications include intrapartum fetal distress, intrauterine growth restriction, oligohydramnios, and in severe cases, stillbirth [3]. Epidemiologic studies have linked approximately 15–20% of all fetal growth restriction and small for gestational age infants to preeclampsia, while 20% of all preterm births are associated with the disease [5,6]. Beyond the obstetric and neonatal consequences, preeclampsia confers long-term risk of complications, including cerebrovascular accident and hypertension [7,8].

An awareness of the syndrome dates back to Hippocrates in ~400 BC, yet preeclampsia remains a significant obstetric concern [12]. According to the World Health Organization, 16% of maternal deaths are attributable to preeclampsia and related gestational hypertensive disorders [13]. Furthermore, multiple studies have displayed an alarming increase in the incidence of gestational hypertension and preeclampsia over the past 3 decades [6,14,15,16]. Combining data from the Agency for Healthcare Research and Quality, the Center for Delivery, Organization, and Markets, Healthcare Cost and Utilization Project, and the National Inpatient Sample, the rate of overall preeclampsia and eclampsia increased by 21% from 2004 to 2014, with the incidence of severe preeclampsia rising by 50% during this period [11]. This imposes a severe clinical and economic burden, as the annual total cost associated with maternal and neonatal consequences of preeclampsia 12 months after delivery was USD 2.18 billion in the United States in 2012 [17].

As clinicians attempt to better manage this rising tide, a number of risk factors have been identified that reflect the complex nature of preeclampsia [15,18]. These include conditions such as chronic hypertension and other classical cardiovascular risk factors, along with chronic renal disease, antiphospholipid syndrome, collagen vascular diseases (e.g., lupus), and preexisting diabetes [18]. Additionally, factors such as nulliparity, a previous diagnosis of preeclampsia, abnormal placentation, multiple gestation, and maternal age at either end of the spectrum (<20 years or >35 years) also increase susceptibility [15]. The rate and severity of preeclampsia are higher among African Americans [19,20], and this likely reflects healthcare inequities as well as a higher incidence of determinants, including chronic hypertension, obesity, and type 2 diabetes, which are underdiagnosed in the African American community [21]. Finally, there appears to be a genetic component to preeclampsia, as a family history of preeclampsia, hypertension, and type II diabetes (either maternal or paternal) also portends higher risk [22,23].

Nonetheless, despite health disparities and the significant clinical impact of preeclampsia, the only “cure” is delivery, and even after childbirth, there remains an elevated risk of cardiovascular and metabolic disease later in life for these mothers and their children [24,25,26,27,28]. Thus, efforts to facilitate early detection, a better understanding of gestational mechanisms, and enhanced treatment modalities are imperative for improved management and health outcomes in patients with this complex condition. As the field advances, there is an increasing awareness that multiple subtypes of preeclampsia exist, and these subtypes may vary in their underlying cause, placental transcriptomic landscape, and disease severity [29,30,31,32]. Proposed classifications include early vs. late onset, in which early is more commonly associated with malplacentation, poor uterine perfusion, and fetal growth restriction. Alternatively, late-onset preeclampsia may be a consequence of placental overgrowth (resulting in compression of the chorionic villi), stress, or senescence towards the end of pregnancy [29,30,31,32]. Redman et al. recently highlighted the notion that regardless of the initiating factors, the placental syncytiotrophoblast layer is susceptible to cell stress throughout pregnancy (i.e., oxidative, mitochondrial, endoplasmic reticulum), and, ultimately, maternal responsiveness to these syncytial stress signals determines whether a woman will develop preeclampsia [29]. Though vascular dysfunction may elicit trophoblast stress or trophoblast stress may disrupt the vasculature, many of the risk factors, proposed mechanisms, and long-term implications of preeclampsia have a direct relationship with the maternal and placental vasculature. Hence, we aim to summarize key avenues of preeclampsia research and highlight the role of the vasculature within these areas.

## 2. Pathophysiology of Preeclampsia

### 2.1. Improper Decidualization and Placentation

It is widely accepted that placental development is disrupted in some pregnancies affected by preeclampsia, leading to cellular, molecular, immunological, and vascular changes [33,34,35], and the role of insufficient decidualization has also received increasing attention [36,37,38,39]. Early-onset preeclampsia is classically thought to be mediated by abnormal placentation and shallow trophoblast invasion within the uterus, thereby resulting in incomplete spiral artery remodeling [33,34,35]. This may lead to placental hypoxia, an aberrant angiogenic state, endothelial dysfunction, further decrements in placental formation, trophoblast stress, and ultimately the maternal presence of preeclampsia [2,35,40]. While much of the etiology remains unknown, research suggests that failed decidual differentiation prior to pregnancy can contribute to impaired trophoblast invasion and its sequela [36,37,38,39].

However, this is a complex syndrome, and the exact order of events in the pathogenesis is unclear. Predicting preeclampsia is imprecise, and determining whether physiological alterations cause preeclampsia or are a secondary result is quite challenging.

#### 2.1.1. Cellular and Molecular Aspects

Decidualization occurs during the mid-secretory phase of the endometrial cycle to prepare the uterus for a potential incoming conceptus [41]. During this process, endometrial stromal cells differentiate into decidual stromal cells [38,39,41]. This process involves morphological alterations and genetic reprogramming to promote tolerance, optimal invasion, angiogenesis, and nutritional support to the embryo preceding placental development [38,39,41]. Research suggesting abnormal decidualization in preeclampsia indicates that human endometrial stromal cells derived from nonpregnant women following severe preeclampsia failed to differentiate, which was evident by a lack of structural changes and an absence of secretory markers [37]. Further, conditioned medium from severe preeclamptic decidual cells was unable to induce cytotrophoblast invasion, unlike culture medium from normal preterm birth decidual cells [37]. One molecular underpinning for this lack of decidualization and inability to stimulate invasion is an annexin A2 deficiency in preeclamptic human endometrial stromal cells [36], which may serve as a critical biomarker in assessing a woman’s susceptibility to developing the disorder [36].

During normal placentation, trophoblasts, which are subdivided into cytotrophoblasts and syncytiotrophoblasts, descend from the blastocyst to form extraembryonic cells critical to placental formation [42,43]. Cytotrophoblasts comprise the inner villous placental layer closest to the fetal circulation, while syncytiotrophoblasts are derived from merging cytotrophoblasts and form the outer villous portion in contact with the maternal environment. Together, this arrangement of cells develops into a branch-like pattern referred to as the chorionic villi [42]. Though chorionic villi are essential for maternal–fetal exchange, the release of placenta-derived syncytiotrophoblast microvilli (STBM) into maternal circulation is elevated in preeclamptic women and impedes endothelial cell proliferation [44]. Furthermore, endothelium-dependent vasodilation is also impaired after infusion of placental STBM vesicles into adipose arterioles obtained during cesarean section. Electron micrographs following this perfusion confirm severe endothelial layer and intracellular organelle disruption, but the underlying smooth muscle remained intact [45]. Thus, the distribution of STBM into the bloodstream is a mechanism of vascular dysfunction in preeclampsia.

Cytotrophoblasts can also differentiate via the extravillous pathway to promote structural remodeling of maternal spiral arteries and sufficient oxygen exchange within the placenta [42,46,47]. In this process, extravillous trophoblasts infiltrate decidual and myometrial tissue during the first trimester to transform the resistance vessels within to those of greater capacitance [42,46,47,48]. Defects in this process during preeclampsia can allow spiral arteries to retain a muscular elastic phenotype, which leads to attenuated oxygen extraction between maternal and fetal circulation and instances of placental hypoxia [43,47].

**Figure 2:**
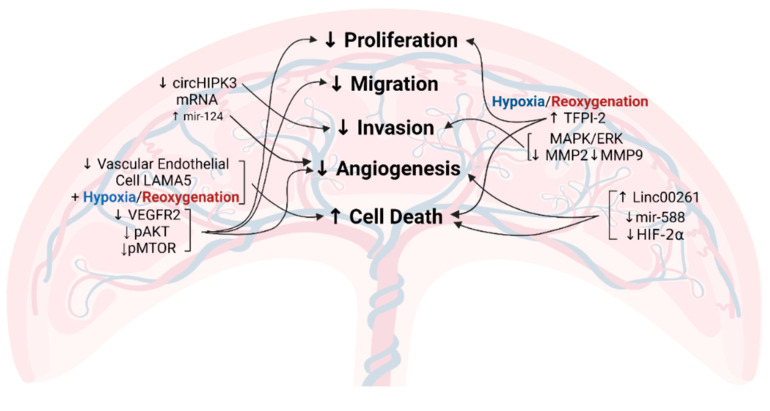
Identified and proposed mechanisms for abnormal placental development in preeclampsia [49,50,51,52,53,54,55,56,57,58,59] (circHIPK3, circular RNA homeodomain interacting protein kinase 3; LAMA5, laminin subunit alpha-5; VEGFR2, vascular endothelial growth factor receptor 2; pAKT, phosphorylated protein kinase B; pMTOR, phosphorylated mammalian target of rapamycin; TFPI-2, tissue factor pathway inhibitor-2; MAPK, mitogen-activated protein kinase; ERK, extracellular-signal-regulated kinase; MMP, matrix metalloproteinase; HIF-2α, hypoxia-induced factor-2α; ↑ refers to upregulation; ↓ refers to downregulation).

Several recent findings indicate novel molecular mechanisms responsible for inadequate placentation [49,50,51,52,53,54,55,56,57,58,59] (Figure 2). 

One proposed contributor involves decreased circular RNA homeodomain interacting protein kinase 3 (circHIPK3) mRNA in preeclamptic women [57]. CircHIPK3 is a circular RNA responsible for impeding microRNA (miRNA) activity [60]. Overexpression in cultured human first-trimester extravillous trophoblast cells facilitated migration and invasion, whereas small interfering RNA (siRNA) attenuated this effect [57]. CircHIPK3 has multiple known miRNA targets, including mir-124 and mir-558 [51,52], and dysregulation of miRNAs is a potential underlying cause for the progression of preeclampsia. It has been reported that mir-124 is upregulated in early-onset preeclampsia [53] and is considered a negative regulator of cardiac angiogenesis [58]. However, the exact role of mir-124 in placental tissue remains unknown. Similarly, the long non-coding RNA Linc00261 is upregulated in preeclampsia, leading to decreased mir-558 [50], which is another target of circHIPK3 [51]. In neuroblastoma cells, mir-558 has been shown to promote growth, invasion, and angiogenesis through the stimulation of hypoxia-induced factor-2α (HIF-2α) [55], so decreased mir-558 may be problematic in the context of preeclampsia if it has a similar role in placental tissue.

There is congruency between factors implicated in preeclampsia and those involved in other hypertensive disorders. In pulmonary hypertension, mir-124 is known to inhibit HIF-2α, initiating alterations in cell proliferation and the complications of this disease [61]. Members of the HIF transcription factor family, including HIF-1α and HIF-2α, have oxygen-sensing capabilities and thereby facilitate downstream regulation of angiogenic factors, including vascular endothelial growth factor (VEGF) and placental growth factor (PlGF) [62,63]. Two placental single-nucleotide polymorphisms in the coding region of *HIF1A* have been associated with preeclampsia as well as elevated HIF-1α activity [64]. HIF-1α also governs trophoblast differentiation during placentation. In a mouse model, transduction of a constitutively active version of HIF-1α into placental trophoblast cells established a preeclamptic-like phenotype, including irregular maternal spiral artery remodeling, altered labyrinth development, and elevated blood pressure [62]. While HIF-1α overactivation as a cause of preeclampsia may seem contradictory to the notion that HIFs are essential for angiogenesis, there is likely an optimal amount of activation, which may be dysregulated in preeclamptic pregnancies. Furthermore, previous studies indicate that different HIF1 isoforms have independent functions, despite overlapping gene targets [63].

Among the many molecular cascades implicated in preeclampsia, the phosphatidylinositol 3-kinase/protein kinase B (PI3K/AKT) and mitogen-activated protein kinase/extracellular-signal-regulated kinase (MAPK/ERK) signaling pathways are essential to trophoblast function and have downstream targets implicated in similar cellular processes, including migration and invasion [56,59,65]. Components of the extracellular matrix are essential for proper placental cell migration [66], and though some cases of preeclampsia were initially thought to be caused by reduced placental cell expansion [67], this deficiency has been linked to vascular dysfunction [56]. Laminin subunit alpha-5 (LAMA5) is a type of laminin, or an extracellular matrix glycoprotein, with decreased expression in vascular endothelial cells of preeclamptic placentas [56]. In human umbilical vein endothelial cells (HUVECs), LAMA5 knockdown combined with exposure to hypoxia/reoxygenation limited proliferation, migration, and angiogenic capacity while increasing cell death [56]. Independently, hypoxia/reperfusion and siLAMA5 caused reduced protein expression of vascular endothelial growth factor receptor 2 (VEGFR2), phosphorylated protein kinase B (pAKT), and phosphorylated mammalian target of rapamycin (pMTOR) (which are both downstream of PI3K), along with increased apoptosis and reduced proliferation [56]. However, the migratory function of trophoblasts relies not only on adhesion molecules such as laminins but also on proteolytic enzymes that degrade the surrounding matrix, called matrix metalloproteinases (MMPs) [68]. Tissue factor pathway inhibitor-2 (TFPI-2), an anticoagulant protein present in the plasma and on the endothelial cell surface, is upregulated in preeclamptic plasma and placental tissue [49,59]. Hypoxia also elevates TFPI-2 and MMP levels [54,59]. *TFPI2* knockdown in human trophoblast cells increased cell invasion and MMP2/9 protein levels via MAPK/ERK signaling [59], which highlights the importance of this pathway and its downstream effects in early processes that may contribute to preeclampsia.

#### 2.1.2. Immunological and Vascular Aspects

The immune system has a profound effect on the maintenance of a healthy pregnancy, as an excessive inflammatory burden can result in the development of preeclampsia [69,70]. Normally, decidual natural killer cells are present in high abundance at the beginning of gestation to stimulate cytokines, MMPs, and angiogenic factors necessary for placental maturation [71,72]. Decidual macrophages have a similar role but also maintain fetal tolerance via immunosuppressive cytokines and phagocytosis of dead trophoblast cells [72]. Additionally, trophoblasts have a direct impact on the vascular arrangement and apoptosis by synthesizing tumor necrosis factor alpha (TNFα), fas-ligand, and other growth factors [73]. In preeclamptic cases, incomplete spiral artery remodeling, placental hypoxia, oxidative damage, and shear stress from uteroplacental blood flow impose a substantial immunological burden. The resulting proinflammatory environment can induce placental apoptosis or necrosis and the release of circulating inflammatory markers [72,74].

Preeclampsia is known to be a state of oxidative stress in which mitochondria are a major source of reactive oxygen species (ROS) [75,76]. Mitochondria-derived free radicals can induce apoptotic caspases and the circulation of cell-free mitochondrial DNA (cf-mtDNA) [76]. Cytosine phosphate guanine (CpG) dinucleotides present in cf-mtDNA are recognized by endosomal toll-like receptor 9 (TLR9) [77], which elicits downstream pro-inflammatory events, including interferon (IFN), MAPK/activator protein-1 (AP-1), and nuclear factor kappa B (NFκB) signaling [78]. Preeclampsia is marked by the release of cf-mtDNA and TLR9 activation [79], but other TLRs are attributed to this disease as well. For instance, administration of lipopolysaccharide, a bacterial endotoxin recognized by TLR4, in pregnant rats precipitated impaired endovascular spiral artery structure, along with the clinical symptoms of preeclampsia [80] (Table 1). 

These inflammatory, blood pressure, and fetal growth effects were alleviated by treatment with a nitric oxide (NO) analog [80], highlighting the theory that vascular dysfunction is likely an etiological factor in preeclampsia. Both TLR4 and TLR9 have suppressive effects on trophoblast migration, suggesting that these receptors are attributed to placental aberrations [82,83]. Placental TLR3 is also upregulated in preeclampsia [87]. Confirming a more causative relationship, treatment with a viral mimetic in pregnant rats resulted in increased placental TLR3 expression, elevated systolic blood pressure, reduced aortic vasodilation, and higher urine protein excretion, and these effects were restricted to pregnant animals [81]. Uterine natural killer cells secrete the anti-inflammatory cytokine interleukin 10 (IL-10), which is highly involved in pregnancy [84,85,88,89,90]. Specific roles of IL-10 include preventing the maternal immune system from rejecting the fetal allograft, decreasing placental endoplasmic reticulum stress, and offsetting antiangiogenic factors [84,85,88,89]. In preeclampsia, there is decreased immunostaining of IL-10 and increased TNFα [91]. In mice, TLR3 activation and *Il10* knockout alone caused preeclamptic phenotypes, and together, these manipulations provoked a more severe state [92]. Exogenous recombinant IL-10 administration restored the impaired endothelium-dependent vasodilatory responses of these mice and may have useful therapeutic potential, considering the limited treatments available [92].

Maternal T cells have many subtypes and a diverse range of immunological functions in pregnancy [72,93]. The cluster of differentiation (CD)4+ class promotes fetal acceptance and consists of regulatory and helper subsets, whereas CD8+ T cells control trophoblast invasion [72]. An adequate ratio of T cell subtypes prevents immune overactivity and detrimental fetal or autoimmune attack [72,94,95]. Regulatory T cells (Tregs) control the defensive actions of T helper cells (Th) [94] and are thought to have imbalanced activity in preeclampsia [96]. More specifically, preeclamptic patients have a suppressed number of Treg cells with upregulated circulating and decidual activity of the proinflammatory Th1 and Th17 subsets [96]. Treg cell depletion in mice causes increased uterine artery vasoconstriction and endothelin-1 production [86], suggesting that altered vasoreactivity in preeclampsia may relate to the reduction in Treg cells.

A successful pregnancy begins with proper placentation, which involves the regulation of many cell types, including trophoblast, immune, and vascular subsets [42,43,46,72]. Placental stress via abnormal placental development or other gestational insults promotes a proinflammatory milieu, accompanied by TLR activation, decreased IL-10, increased TNFα, changes in T cell ratios, and disrupted vasodilatory function [79,80,82,83,91,92,96]. It is probable that immunological and vascular anomalies are present before the onset of maternal preeclamptic symptoms and are further exacerbated throughout its progression.

## 3. Circulating and Placenta-Derived Vascular Substances Associated with Preeclampsia (VEGF, PlGF, sFLT-1, ENG, and sENG)

Preeclampsia is characterized by an imbalance of pro- and antiangiogenic factors, which directly impacts endothelial function [97,98,99,100]. VEGFA stimulates angiogenesis, vascular permeability, and cell migration by binding to its tyrosine kinase receptors VEGFR1 and VEGFR2 [101,102]. VEGFA binding to VEGFR2 elicits stronger signaling than VEGFR1 [103] via activation of the phospholipase C gamma (PLCy)/protein kinase C (PKC)/MAPK pathway involved in endothelial cell proliferation [101]. During placental villous development, VEGFA is present in trophoblasts and perivascular cells to support de novo vascular development (i.e., vasculogenesis), as well as vessel expansion via endothelial sprouting (i.e., angiogenesis) [104]. In pregnancy, VEGF induces a more robust activation of endothelial nitric oxide synthase (eNOS), with NO production primarily occurring through VEGFR2-induced PI3K/AKT signaling [105,106]. PlGF, its proangiogenic counterpart, binds to VEGFR1 to increase the likelihood of VEGF binding to VEGFR-2 [107]. PlGF’s interaction with VEGFR1 also promotes other critical events, such as transphosphorylation of VEGFR2, augmenting its downstream signaling cascade [107]. Similar to VEGF, the actions of PlGF facilitate the growth and migration of endothelial and trophoblast cells [107,108,109]. In healthy pregnancies, PlGF increases until 32 weeks and then subsequently declines [110,111]. However, in preeclampsia, there is a marked reduction in venous levels as early as 13–16 weeks, occurring before the onset of other clinical symptoms [108,112]. Not only does this have adverse cardiovascular consequences during pregnancy, but these vascular pathologies and unfavorable cardiac remodeling can persist for years beyond pregnancy [113]. This suggests that though overt symptoms are often resolved after delivery, preeclamptic mothers are at risk for cardiovascular disease years or decades later.

While the VEGFR is membrane bound, a truncated, soluble version known as soluble fms-like tyrosine kinase 1 (sFLT-1) sequesters ligands that bind to the VEGFR, specifically VEGF and PlGF [114]. sFLT-1 is similar to VEGFR1 but lacks the membrane-spanning domain [115]. sFLT-1 plays a role in the pathogenesis of preeclampsia and can be detected in the serum and placenta before other clinical manifestations, including proteinuria and hypertension [99,116]. The sFLT-1/PlGF ratio from 24 weeks to 36 weeks 6 days has been deemed a useful tool for predicting the absence of preeclampsia within 1 week of measurement, with a negative predictive value of 99.3% at a cutoff of 38 [117]. However, the positive predictive value for the diagnosis of preeclampsia within the next 4 weeks at this threshold was only 36.7% [117]. Thus, while this test is informative, earlier detection methods and tools with greater positive predictive value would be more powerful for improving the care of women prior to preeclampsia.

Adding to the complexity of this disorder, it has been discovered that a unique isoform of sFLT-1 predominates after the first trimester in both healthy and preeclamptic pregnancies [115,118]. At term, preeclamptic placentas express much higher *sFLT1* mRNA, specifically in syncytial knots, which are a source of circulating sFLT [118]. Furthermore, hypoxic conditions stimulate mRNA expression of the novel *sFLT1* variant and sFLT-1 peptide release in cultured primary cytotrophoblast and syncytiotrophoblast cells [115,119], but these effects are cell-specific, such that low oxygen tension does not increase the release of sFLT-1 protein in the culture medium of HUVECS or villous fibroblasts [119]. In cytotrophoblasts, no changes in free VEGF protein were detected under conditions of reduced oxygen tension despite an increase in total VEGF protein production, suggesting sequestration by sFLT-1 [119]. This imbalance leads to elevated oxidative stress, evident by increased lipid peroxidase relative to superoxide dismutase [120], a key antioxidant for scavenging oxygen free radicals [121].

Endoglin (ENG) is yet another hypoxia-induced protein implicated in preeclampsia [54,122,123]. This protein is located on endothelial cells, syncytiotrophoblasts, and columnar cytotrophoblasts prior to uterine invasion and exists in either a proangiogenic membrane-bound form or an antiangiogenic soluble form [54,122,124]. ENG serves as a receptor for the cytokines transforming growth factor-beta 1 and 3 (TGF-β1 and TGF-β3), which perform functions related to cell proliferation and apoptosis [122,125,126]. There are contradictory findings regarding the role of TGF-β in preeclampsia, which may be a product of gestational-age-related variations. Ayatollahi et al. reported no differences in serum TGF-β1 between healthy and preeclamptic pregnancies at 36–40 weeks and suggested that the anti-inflammatory properties of TGF-β may be instrumental for fetal-allograft survival [127]. Alam et al. found elevated TGF-β1 levels in preeclamptic patients from 30 to 33 weeks with a slight drop after 33 weeks [125], which may explain the lack of difference in the aforementioned study. One proposed explanation for the increase in TGF-β1 involves the shedding of necrotic placental trophoblast cells into the circulation, which are thought to undergo apoptosis in healthy pregnancies. When necrotic trophoblasts are phagocytosed by endothelial cells, the endothelial cells secrete TGF-β1, leading to IL-6 release. This causes placental soluble endoglin (sENG) secretion, sENG occupancy of ENG receptors, and impaired NO synthesis. Because TGF-β cannot bind to its receptor, its circulatory presence is increased, explaining the rise in TGF-β in preeclamptic women [125].

**Figure 3:**
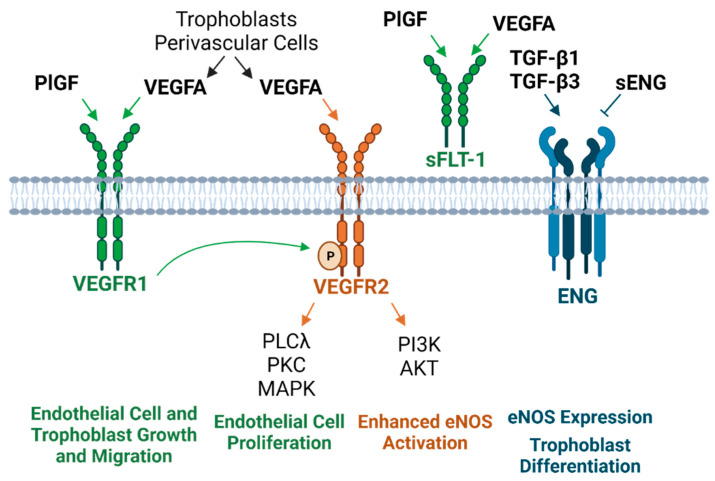
Circulating and placenta-derived vascular substances associated with preeclampsia and their downstream cellular effects [54,101,102,104,105,106,107,114,115,122,123,125,126,130,131,133]. PlGF, VEGFA, and ENG are considered “proangiogenic” factors, whereas sFLT-1 and ENG are “antiangiogenic.” sFLT-1 sequesters PlGF and VEGFA, and sENG blocks TGF-β binding to the ENG receptor (PlGF, placental growth factor; VEGFA, vascular endothelial growth factor A; sFLT-1, soluble fms-like tyrosine kinase 1; ENG, endoglin; sENG, soluble endoglin; VEGFR1 and VEGFR2, vascular endothelial growth factor receptor 1 and 2; TGF-β, transforming growth factor-beta; PLCλ, phospholipase C gamma; PKC, protein kinase C; MAPK, mitogen-activated protein kinase; PI3K, phosphatidylinositol 3-kinase; AKT, protein kinase B; eNOS, endothelial nitric oxide synthase).

Both membrane and sENG are elevated in preeclampsia and have been considered as a diagnostic tool for detecting the syndrome [123,128]. Excess sENG has detrimental effects on endothelial tube development. In animal models, injection of both sENG and sFLT-1 results in a severe preeclamptic phenotype, accompanied by liver defects, fetal weight restriction, and neurological impairments [129]. More favorably, transmembrane ENG has been associated with NO-mediated vasodilation via eNOS expression [130,131] as well as trophoblast differentiation prior to invasion [126,130] (Figure 3). However, fluctuations in membrane ENG concentration and localization throughout pregnancy may be necessary for the maintenance of placental function because *ENG* knockdown has been shown to support extravillous trophoblast invasion [132].

Several hypotheses exist regarding the mechanism of elevated sENG in preeclampsia, many of which are thought to independently contribute to the disorder [54,130]. Proposed notions include insufficient endogenous oxidative protective factors, NFκB activation, the MAPK stress response, MMP-regulated cleavage from endothelial plasma membranes, and angiotensin II type 1 receptor autoantibodies (AT1-AA) [54,130]. Experimental AT1A receptor activation elicits many preeclamptic-like phenotypes, including cell proliferation, vasoconstriction, renal fluid reabsorption, and vascular smooth muscle cell (VSMC) hypertrophy [134]. Mechanistically, rat models suggest that reduced uterine perfusion pressure stimulates increases in TNFα, which is coupled to AT1AA production [135,136]. Further, AT1AA infusion induced elevations in sENG as well as endothelin-1, and sFLT-1 [137,138].

While there are no reliable preventive or treatment therapeutics for preeclampsia [139,140,141], numerous studies support the notion that proangiogenic factors may be a viable future target against the high sFLT-1 levels found in preeclampsia [99,142,143,144]. Administration of VEGF and PlGF reestablished endothelial tube formation in HUVECs after it was impeded by preeclamptic serum [99]. In vitro, VEGF and PlGF produce dose-dependent arteriole vasodilation, and this response is blocked by sFLT1 [99]. Along with compromised vasomotor function, sFLT-1 increases placental and vascular superoxide production, and vasodilatory impairment can be reversed by free-radical scavenging [145]. In vivo, animal models indicate that PlGF infusion eliminates hypertension caused by both sFLT-1 and reduced uteroplacental perfusion [142,143]. Removal of sFLT-1 from women prolonged pregnancy [144]. Therefore, reversing the ratio of circulating vascular substances in preeclampsia has the potential to restore the dysregulated homeostatic state of preeclamptic women or prevent the progression of this syndrome in those at high risk.

## 4. Endothelial Damage in Preeclampsia

The endothelium, or inner layer of blood vessels, serves a wide array of functions that encompass but are not constrained to hemostasis, fibrinolysis, regulation of vascular tone, mediation of inflammatory cascades, and permeability [146,147,148,149]. Endothelial dysfunction, specifically in the form of barrier disruption and impaired vasodilatory capacity, is prevalent in preeclampsia and implicated in many stages of the disease [150,151,152,153,154]. Hemodynamic shifts accompanying compromised endothelial junction integrity, specifically those related to vasopressin, may precede early malfunctions in placental development [155], or in late-onset preeclampsia, endothelial damage may render a mother unable to buffer trophoblast-derived stress signals that accumulate throughout pregnancy [29,32]. The vascular defects of late-stage preeclampsia appear to be targeted to the endothelium in certain vascular beds, which is evident by the incubation of uterine myometrial resistance vessels with preeclamptic plasma [152]. Preeclamptic plasma restricted endothelium-dependent relaxation, but the effect only occurred with an intact endothelium, demonstrating that the vascular smooth muscle was unimpaired [152]. Similarly, placental vessels obtained from preeclamptic pregnancies show attenuated endothelial function with unaltered smooth muscle-mediated dilation [153]. These endothelial-specific findings have also been confirmed in vivo, as endothelium-dependent flow-mediated dilation was impaired in women with a history of preeclampsia compared to those without [154]. Together, the imbalance between constriction and relaxation and hemodynamic modifications that alter body fluid homeostasis are prominent features of preeclampsia [156].

### 4.1. Volemic Changes Associated with Endothelial Barrier Integrity

Under normal physiologic conditions, pregnancy is accompanied by systemic arterial vasodilation that is countered by an increase in cardiac output, sympathetic activation, stimulation of the renin–angiotensin–aldosterone system, and non-osmotic vasopressin release [157,158]. As a result, maternal plasma volume expansion begins in the first trimester, resulting in a subsequent decrease in plasma osmolality, and this expansion continues until around 32 weeks [159]. The maintenance of maternal blood volume is essential for fetal development [158], and overall blood and plasma volumes are reduced in those with preeclampsia despite elevations in blood pressure [160,161]. In these hypovolemic women, body fluid is distributed more to the interstitial space rather than the intravascular space, which is indicative of capillary leak [162,163,164] and paralleled in the deoxycorticosterone acid salt rat model of preeclampsia [165]. Proposed mechanisms of disrupted endothelial barrier integrity include the release of placenta-derived HtrA serine peptidase 4 (HTRA4), which is increased in serum from patients with early-onset preeclampsia and responsible for cleaving VE-cadherin, an endothelial junctional protein [150,166]. In vitro incubation of HUVECs with similar levels of HTRA4 altered cell morphology and the integrity of cell junctions [150]. Likewise, the expression of chymotrypsin-like protease is increased in the vascular endothelium of women diagnosed with preeclampsia and promotes VE-cadherin disruption and endothelial permeability via its activation of protease-activated receptor 2 (PAR-2) [167,168].

The presence of hypovolemia within the vessels due to endothelial leak shifts the plasma osmolality vs. vasopressin relationship such that plasma vasopressin levels are higher at a given osmolality [169,170]. Santillan et al. demonstrated that plasma copeptin, the pro-segment of vasopressin, is elevated as early as the 6th week of pregnancy in women prior to the onset of preeclampsia, independent of confounding variables such as maternal age and body mass index [171]. With a cutoff value of 811 pg/mL in the first trimester, this test has a sensitivity of 88% and a specificity of 81%. Even within the third trimester, a threshold of 758 pg/mL maintains a sensitivity and specificity of 78% and 71%, respectively [171]. Further, a causative relationship between vasopressin and preeclampsia was established in a mouse model, where infusion of vasopressin had no effect prior to pregnancy but then led to hypertension, renal glomerular endotheliosis, spiral artery malformation, decreased PlGF, and elevated placental oxidative markers during gestation [155]. In conclusion, increased vasopressin during preeclampsia may be a result of hypovolemia or another mechanism, such as a hypothalamic disturbance; regardless, the secretion of this hormone elicits many maternal and placental phenotypes typical of this syndrome and may be used as an early biomarker [155,171].

### 4.2. Altered Vasomotor Tone

To offset pregnancy-induced increases in blood volume, compensatory alterations include decreased sensitivity to vasoconstrictive hormones, along with increased production and responsiveness to vasodilators such as NO and prostaglandins [156,172].

However, studies have demonstrated that NO availability is diminished in preeclamptic women [173,174]. NO is a prominent endothelium-derived vasodilator catalyzed by the conversion of L-arginine to L-citrulline via the enzyme eNOS [148,175]. Its release is mediated by acetylcholine, bradykinin, mechanical shear stress, and a variety of other stimuli [148]. When NO is synthesized, it diffuses from the endothelium to the surrounding vascular smooth muscle cells. This facilitates vasodilation through a cascade of events involving the formation of cyclic guanosine monophosphate, activation of protein kinase G, increased potassium channel conductance, hyperpolarization of the smooth muscle sarcolemma, and decreased cytosolic calcium content [148,175,176]. eNOS is a calcium calmodulin-dependent enzyme; hence, its activation is heavily reliant on calcium [177]. Additionally, other eNOS modifications, including phosphorylation, render it more sensitive to calcium signaling [177], but phosphorylation alone is not adequate for eNOS activation in pregnant uterine artery endothelial cells [178].

Research indicates that augmented eNOS activation during pregnancy is mediated by adaptive signaling mechanisms to permit calcium influx, which are deficient in preeclampsia [179]. This calcium entry occurs through transient receptor potential channels and is maintained only with proper connexin 43 (Cx43) gap junction communication [179,180]. Interestingly, the most common variant of VEGFA, referred to as VEGF165, has been shown to initially facilitate NO generation in endothelial cells via elevated calcium responses [176]. However, longer exposure to VEGF165 impairs calcium-provoked vasodilation, presumably by promoting Cx43 phosphorylation, which attenuates calcium signaling in these cells [176,180]. VEGF has also been shown to facilitate the phosphorylation of eNOS on serine 1177 in glomerular endothelial cells, which is necessary for eNOS activation [181]. In summary, VEGF, which is sequestered by high levels of sFLT-1 in preeclampsia, promotes both calcium-dependent and -independent (i.e., phosphorylation) stimulation of eNOS, whereas vasodilators such as acetylcholine signal through the Gαq pathway to increase endothelial calcium and activate eNOS [182].

NO is also positively regulated by the endogenous vasodilator hydrogen sulfide [183,184,185,186,187], and both of these gaseous molecules work in concert [185]. Though not specific to preeclampsia, hydrogen sulfide enhances NO function by impeding cGMP degradation [185], activating eNOS via a PI3K/Akt-induced phosphorylation event [185], facilitating mir-455-3p and subsequent eNOS expression [186], and arbitrating the reduction of nitrite to NO through xanthine oxidase [187]. Conversely, NO is also necessary for the maximal vasodilatory and angiogenic effects of hydrogen sulfide [185]. Instances of both lowered and elevated plasma hydrogen sulfide levels have been reported in preeclampsia [188,189,190]. This molecule can be separately synthesized by cystathionine β synthase (CBS), cystathionine γ lyase (CSE), and 3-mercaptopyruvate sulfurtransferase (MPST) [191]. Within the placenta, CBS and CSE proteins were localized to fetal endothelial cells [192], and decreases in placental mRNA expression of CBS [192] and CSE [189] have been documented in preeclampsia. Administration of a CSE inhibitor beginning at gestational day 8.5 in mice led to hypertension, altered placental labyrinth vascular patterning, and fetal growth restriction, which was restored by hydrogen sulfide, establishing the direct significance of hydrogen sulfide in preeclampsia [189]. However, it should be noted that just as there is crosstalk among angiogenic, NO, and migration pathways [105,106,107,108,109], a similar relationship is paralleled by hydrogen sulfide [193]. Cell culture models indicate that CSE deficiency has a negative impact on trophoblast invasion and propagates antiangiogenic factor release [189], which, at least in part, may be responsible for the physiological effects exhibited by CSE inhibition in pregnant mice [189].

Oxidative stress in preeclampsia may be linked to the nitric oxide pathway [194,195]. For instance, arginase is upregulated in preeclamptic plasma and competes for L-arginine, the substrate used by NOS [194]. Similarly, circulating maternal asymmetric dimethylarginine (ADMA), a competitive inhibitor of arginine binding to NOS, is elevated mid-gestation and at the time of delivery in women with a diagnosis of preeclampsia [195,196]. Both arginase and ADMA result in the formation of superoxide [194,195], and exposing HUVECs to preeclamptic plasma also elevates oxidative markers, including superoxide [197]. Human data confirm that scavenging free radicals using ascorbic acid restores endothelium-dependent vasodilation [154].

In addition to vasodilatory substances such as nitric oxide, the vasoconstrictive peptides angiotensin II and endothelin-1 also play a role in the symptomology of preeclampsia [198,199]. While angiotensin II exerts contractive effects on vascular smooth muscle cells, it can also act on endothelial cells, prompting prostacyclin synthesis and endothelin mRNA activation [200]. During pregnancy, women with preeclampsia have increased angiotensin II sensitivity and decreased circulating levels [201]. The increased sensitivity to angiotensin II persists postpartum, along with microvascular dysfunction, and angiotensin II receptor blockers facilitate vasodilation in this cohort [202].

Endothelin-1 is secreted from endothelial cells and is a strong regulator of vasomotor tone through its interactions with endothelin-A (ETA) and endothelin-B (ETB) receptors [203]. ETA receptors are localized to smooth muscle cells and evoke contraction, whereas ETB receptors are present on both the endothelial and smooth muscle vascular layers and elicit differential responses in each: contraction in smooth muscle and vasodilation in the endothelium [204]. In the context of preeclampsia, endothelin-1 is a powerful regulator of uterine artery resistance, which has a direct impact on uteroplacental perfusion [205,206]. Pharmacological antagonism of the ETA receptor blunted the rise in mean arterial pressure in a rat model of uterine ischemia, with no effect in nonpregnant animals [207]. After testing the effects of different classes of antihypertensive medications, dihydropyridines (a type of calcium channel blocker) were the most effective in blocking and reversing endothelin-1-mediated constriction in human uterine arteries obtained after hysterectomy [205]. More specifically, ex vivo pretreatment with these calcium channel antagonists in endothelium-denuded vessels reduced tension and maximal contraction to endothelin-1, even at high doses [205]. While a combination of selective ETA and ETB receptor antagonists did decrease contractile responses to endothelin-1 at lower concentrations, these compounds had no effect under conditions of high endothehlin-1, suggesting that dihydropyridines may be the most useful for enhancing blood supply to the placenta [205]. However, the function of endothelin-1 extends far beyond vasoconstriction and can also induce inflammation, angiogenesis, VSMC proliferation, and vasodilation, depending on the receptor subtype being activated [208].

Angiotensin II, endothelin-1, and other vasoconstrictive hormones, including vasopressin, predominantly signal through G protein-coupled receptors (GPCRs) [209,210], and excess input from these substances has been attributed to the symptomology of preeclampsia [155,171,198,199]. Regulator of G protein signaling (RGS) proteins dampen the strength and duration of GPCR activation by hydrolyzing the guanosine-5′-triphosphate bound to an active Gα subunit [211,212,213,214]. Of the large family of RGS proteins, RGS2 is of particular importance in buffering this GPCR signaling associated with preeclampsia [213,215] and is present in vascular cells, including endothelial cells [216], as well as many other cell types of the placenta, including trophoblasts, immune cells, and fibroblasts [213,217]. Recently, Perschbacher et al. found that *RGS2* was decreased in the placenta during human preeclampsia, and heterozygous knockout in only the fetoplacental unit of mice was sufficient to cause key hallmarks of the disorder, such as diastolic hypertension and proteinuria [213]. Further, transcriptomic analyses revealed that there was overlap in molecular pathways enriched in human preeclamptic placenta and mice with disrupted *Rgs2*, including those related to mitochondrial dysfunction, the unfolded protein response, and oxidative stress [213]. Regarding the maternal vasculature and complementing this work, Koch et al. found that elimination of *Rgs2* in mice attenuated uterine artery blood flow and increased the resistive index during mid-gestation [215]. Hence, these studies indicate that preeclampsia may also be a result of disinhibition of GPCR signaling to various hormones, which contribute to placental and vascular dysfunction [213,215].

Overall, the pathogenic vascular state of preeclampsia can be attributed to the decreased synthesis of relaxing substances and elevated vasoconstrictive signaling [218,219]. This signaling may be a consequence of increased abundance or sensitivity to vasoconstrictive hormones as well as disinhibition of these pathways [213,215,218,219]. Vascular resistance has profound effects, both systemically and on specific vascular beds, and has been demonstrated experimentally in vivo, ex vivo, and in vitro [152,154,197,205,207]. Systemically, previously preeclamptic women display impaired endothelium-dependent vasodilation, which can be ameliorated by angiotensin II receptor blockers [154,202]. Additionally, diminished uteroplacental perfusion secondary to elevations in mean arterial pressure is prevented by ETA antagonism in rats [207]. Both uterine and placental arteries displayed attenuated endothelium-dependent vasodilatory function in the context of preeclampsia [152,153], which may be partially caused by oxidative stress [154,197]. In combination, uteroplacental resistance negatively impacts fetoplacental exchange, while systemic resistance can contribute to glomerular endotheliosis, liver failure, and central nervous system damage. Together, this creates an array of multiorgan dysfunctions [99,220,221].

## 5. Preeclampsia-Associated Platelet Alterations

**Figure 4:**
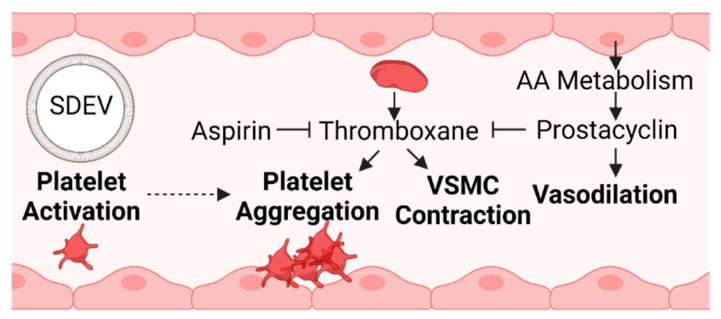
An imbalance in coagulation factors promotes a more prothrombotic environment during preeclampsia [226,227,228]. Prostacyclin is a vasodilatory product of arachidonic acid metabolism derived from endothelial cells [225]. Prostacyclin and aspirin oppose the actions of thromboxane, and the ratio of prostacyclin to thromboxane decreases in preeclampsia [226,229]. The accumulation of placental stress can lead to the release of SDEVs into maternal circulation. SDEVs can promote platelet activation, a precursory step to platelet aggregation and the formation of blood clots [29,230] (SDEV, syncytiotrophoblast-derived extracellular vesicle; VSMC, vascular smooth muscle cell; AA, arachidonic acid).

Trophoblast stress is a common feature of preeclampsia and results in a compensatory surge of inflammatory mediators [29,75,222,223,224]. Of these, prostacyclin is a product of arachidonic acid metabolism [224,225]. Its role as a vasodilatory factor opposes the action of thromboxane, which elicits vascular smooth muscle constriction and platelet aggregation [226] (Figure 4). 

These two vasoactive substances are produced by the endothelium, platelets, and reproductive tissues, and the balance between them is disrupted in preeclampsia, with the level of placental and plasma thromboxane exceeding that of prostacyclin [226,227,228]. The pharmacological effects of aspirin diminish platelet thromboxane production, altering the prostaglandin thromboxane ratio [227]. A meta-analysis revealed that starting low-dose aspirin early in pregnancy as a preventative measure for preeclampsia has moderately favorable results [231]. The American College of Obstetricians and Gynecologists, the Society for Maternal-Fetal Medicine, and the U.S. Preventative Services Task Force now recommend low-dose aspirin for women with a high risk of preeclampsia [232].

Platelet activation, aggregation, and blood coagulation (clotting) are interrelated processes [233]. Briefly, platelets have adhesive properties and, upon binding to an injured endothelium, release substances such as thromboxane to promote aggregation. Platelet aggregation encourages the formation of a platelet plug and thrombin-mediated generation of a cross-linked fibrin clot [233]. A recent systemic review and meta-analysis suggests that preeclamptic patients have higher mean platelet volume (indicating platelet activation) and a higher likelihood of adhesion and aggregation [234]. In this paper, Jakobsen et al. reported inconsistent findings regarding aggregation, with more studies suggesting no difference or decreased aggregation, but these particular studies did not assess adhesion [234]. One study that did assess platelet adhesion reported decreased immunohistochemical expression of platelet endothelial cell adhesion molecule-1 and increased intercellular adhesion molecule-1 in the human placenta of preeclamptic individuals, which has been proposed to play a role in trophoblast invasion and vascular dysfunction [234,235]. Converging the idea that syncytiotrophoblast stress is a final common factor that leads to the maternal elements of preeclampsia [29] with the importance of platelet function in this syndrome, syncytiotrophoblast-derived extracellular vesicles (SDEVs) have been shown to activate platelets ex vivo [230]. SDEVs obtained from preeclamptic placentas evoke greater platelet activation than those from normal pregnancies, but platelet aggregation is prevented by aspirin treatment [230].

During pregnancy, there is a natural decline in platelet count throughout gestation [236], part of which may be attributed to sequestration of blood cells in the intervillous space [237], increases in plasma volume [238], and heightened aggregation from thromboxane A2 [239]. Thrombocytopenia beyond normal pregnancy-induced platelet decreases is commonly seen in preeclampsia and may specifically be accompanied by reduced platelet numbers [239,240,241] and activated coagulation [240,242]. Together, these hemostatic effects contribute to bleeding and microthrombi risk in preeclamptic mothers [239,243].

## 6. Oxidative Stress, Mitochondrial DNA Damage, and TLR9 Activation

The generation of reactive oxygen species via placental hypoxia, immune activation, and other cellular insults has numerous consequences, including mitochondrial DNA (mtDNA) damage [244,245,246,247]. The DNA repair capabilities of the mitochondria are less extensive than those for nuclear DNA, which renders cells with mtDNA mutations more susceptible to death by apoptosis or necrosis [248,249]. This causes the release of DNA into the maternal circulation, which is considered a damage-associated molecular pattern (DAMP), recognized by pattern recognition receptors such as TLR9 [250]. TLR9 is a pro-inflammatory innate immune component activated by hypomethylated CpG dinucleotides, which are prevalent in mtDNA and bacteria [251,252]. Although surface receptors are also present, the recognition of DNA by TLR9 primarily occurs in endolysosomes because the acidic environment allows TLR9 to more easily bind to negative DNA [251]. Thus, DNA enters the cell through endocytosis [253] and, upon TLR9 binding, causes a downstream proinflammatory cascade of events, including signaling for IFNs, NFκB, and AP-1 [83].

**Figure 5:**
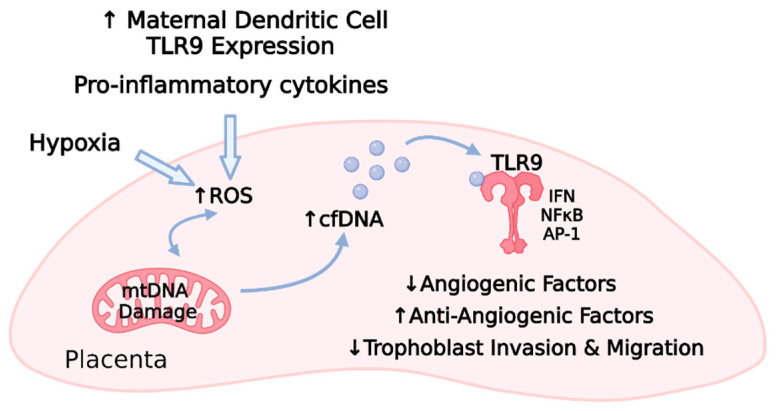
Maternal factors, including TLR9-induced proinflammatory cytokine release and placental hypoxia, promote a cascade of oxidative stress, mitochondrial DNA damage, cell-free DNA release, TLR9 activation, and subsequent TLR9 activation within the placenta [83,244,245,246,247,248,249,250,251,252,254,255]. Animal and cell culture models indicate that TLR9-related signaling results in decreased angiogenic factors, increased antiangiogenic factors, and impaired trophoblast function [83] (TLR9, toll-like receptor 9; ROS, reactive oxygen species; mtDNA, mitochondrial DNA; cfDNA, cell-free DNA; IFN, interferon; NFκB, nuclear factor kappa B; AP-1, activator protein-1; ↑ refers to upregulation; ↓ refers to downregulation).

Supporting these notions, there is an increased abundance of serum mtDNA in preeclamptic plasma [254], and TLR9 activity is elevated upon the presentation of preeclamptic symptoms [247] (Figure 5). Recent findings by He et al. link the TLR9 inflammatory response to other aspects of preeclampsia, including angiogenesis and trophoblast function [83]. In this study, human placental VEGFA was decreased, but TLR9 and sFLT-1 were increased in preeclamptic samples [83]. Applying these findings to a mouse model, a TLR9 agonist induced the traditional hallmarks of preeclampsia and also reproduced the downregulated VEGFA and elevated sFLT-1 observed in human tissue [83]. siRNA knockdown of *TLR9* in human trophoblast cells facilitated migration and invasion, which highlights the importance of TLR9 in early phases of placentation as well [83]. The dendritic cells of preeclamptic women appear to be hyperresponsive to immune-evoking substances, suggesting a potential source for this excess TLR9 engagement. In preeclampsia, dendritic cell TLR9 expression levels were higher, and upon stimulation, these receptors evoked more proinflammatory cytokines compared to healthy pregnant controls [255]. These data reveal an interconnected relationship among inflammation, oxidative stress, TLR9 activation, the release of antiangiogenic factors, and trophoblast dysfunction [83,244,245,248,249,250] and reiterate the complex interplay between many molecular mediators in women with preeclampsia. Understanding each of these components and how they interact is essential to mitigating the disorder but has proven difficult considering its heterogeneity and the lack of a single, well-understood initiating mechanism [29,31].

## 7. Conclusions

Despite the profound detrimental impact that preeclampsia has upon maternal and fetal health, its pathogenesis has yet to be definitively determined and likely varies [30,256], which has limited the development of treatment options [256]. Thus, management of preeclampsia has been primarily symptomatic, focusing on maintaining acceptable blood pressure ranges, neuroprotection, and seizure prophylaxis, with prompt delivery at term or 34 weeks for cases with severe features [1]. However, it has been established that instances of vascular impairments are evident in all stages of preeclampsia, beginning with placentation and extending well beyond delivery [7,202], and are likely a product of some combination of insufficient trophoblast invasion, poor placental oxygen extraction, a proinflammatory immune environment, antiangiogenic factors, endothelial dysfunction, and oxidative stress [34,100,135,257,258,259].

Due to a lack of robust research assessing vascular indices prior to pregnancy and preceding the onset of preeclampsia, it is unclear whether women who develop this syndrome have an underlying vascular pathology [260,261] or if the detrimental vascular effects are solely a byproduct of exacerbated trophoblast stress signals to the mother [262]. It is likely that both aspects play a role, and the dysregulated physiological state of preeclampsia begins well before the clinical diagnosis. Therefore, advancing early detection methods and screening tools is of critical importance. Though not yet utilized in the clinic, routinely measuring vasopressin during pregnancy is a promising avenue for predicting the future development of preeclampsia and providing more proactive care in these patients [171]. In terms of molecular targets, less explored areas include the modulation of RGS proteins to mitigate the negative effects of excessive GPCR induction via hormones such as angiotensin II, endothelin-1, and vasopressin [213,263] or the alleviation of cellular stress that leads to mitochondrial dysfunction, cell death, circulating DNA, and subsequent TLR9 activation [79,83,244,247,252,264,265,266]. Though much remains undiscovered, translational research [152,153,154], basic animal models [155,213,215], and mechanistic cell work [56,57,150,213] have made a profound impact in the field thus far, and emerging technologies such as trophoblast organoid cultures [267] provide great potential for new insight. Thus, collaborating across the spectrum, from bench to bedside, will allow the most rapid acceleration in our understanding of preeclampsia and may foster the development of novel targeted therapeutics.

## Figures and Tables

**Table 1:** Immune components altered in human preeclampsia and the effects of manipulation in cell culture and animal models.

Preeclampsia	Physiological Effects	Reference
↑ TLR3	Elevated systolic blood pressure	[81]
	Reduced aortic vasodilation	[81]
	Increased urinary protein concentrations	[81]
↑ TLR4	Impaired endovascular spiral artery structure	[80]
	Suppressed trophoblast migration	[82]
↑ TLR9	Suppressed trophoblast migration	[83]
	Decreased VEGFA, increased sFLT-1	[83]
↓ IL-10	Decreased buffering of antiangiogenic factors	[84]
	Increased endoplasmic reticulum stress	[85]
↓ Treg cells	Increased uterine artery vasoconstriction	[86]
	Elevated endothelin-1 production	[86]

TLR, toll-like receptor; VEGFA, vascular endothelial growth factor A; sFLT-1, soluble fms-like tyrosine kinase 1; IL-10, interleukin 10; Treg, regulatory T cell; ↑ refers to upregulation; ↓ refers to downregulation.

## Data Availability

Not applicable.
